# Aspirin and cancer survival: a systematic review and meta-analyses of 118 observational studies of aspirin and 18 cancers

**DOI:** 10.3332/ecancer.2021.1258

**Published:** 2021-07-02

**Authors:** Peter C Elwood, Gareth Morgan, Christine Delon, Majd Protty, Julieta Galante, Janet Pickering, John Watkins, Alison Weightman, Delyth Morris

**Affiliations:** 1 Division of Population Medicine, Cardiff University, Cardiff CF14 4XN, UK; 2 Freelance statistician, London, UK; 3 Cardiff Lipidomics Group, Cardiff University, UK; 4 University of Cambridge, Cambridge, UK; 5 National Institute for Health Research (NIHR) Applied Research Collaboration East of England, Cambridge, UK; 6 Public Health Wales, Cardiff, UK; 7 Specialist Unit for Review Evidence, Cardiff University, Cardiff, UK; 8 University Library Service, Cardiff University, Cardiff, UK

**Keywords:** aspirin, cancer, survival, mortality, bleeding, thromboembolism

## Abstract

**Background:**

Despite the accumulation of research papers on aspirin and cancer, there is doubt as to whether or not aspirin is an acceptable and effective adjunct treatment of cancer. The results of several randomised trials are awaited, and these should give clear evidence on three common cancers: colon, breast and prostate. The biological effects of aspirin appear likely however to be of relevance to cancer generally, and to metastatic spread, rather than just to one or a few cancers, and there is already a lot of evidence, mainly from observational studies, on the association between aspirin and survival in a wide range of cancers.

**Aims:**

In order to test the hypothesis that aspirin taking is associated with an increase in the survival of patients with cancer, we conducted a series of systematic literature searches to identify clinical studies of patients with cancer, some of whom took aspirin after having received a diagnosis of cancer.

**Results:**

Three literature searches identified 118 published observational studies in patients with 18 different cancers. Eighty-one studies report on aspirin and cancer mortality and 63 studies report on all-cause mortality. Within a total of about a quarter of a million patients with cancer who reported taking aspirin, representing 20%–25% of the total cohort, we found aspirin to be associated with a reduction of about 20% in cancer deaths (pooled hazard ratio (HR): 0.79; 95% confidence intervals: 0.73, 0.84 in 70 reports and a pooled odds ratio (OR): 0.67; 0.45, 1.00 in 11 reports) with similar reductions in all-cause mortality (HR: 0.80; 0.74, 0.86 in 56 studies and OR: 0.57; 0.36, 0.89 in seven studies). The relative safety of aspirin taking was examined in the studies and the corresponding author of every paper was written to asking for additional information on bleeding. As expected, the frequency of bleeding increased in the patients taking aspirin, but fatal bleeding was rare and no author reported a significant excess in fatal bleeds associated with aspirin. No author mentioned cerebral bleeding in the patients they had followed.

**Conclusions:**

There is a considerable body of evidence suggestive of about a 20% reduction in mortality in patients with cancer who take aspirin, and the benefit appears not to be restricted to one or a few cancers. Aspirin, therefore, appears to deserve serious consideration as an adjuvant treatment of cancer, and patients with cancer, and their carers, have a right to be informed of the available evidence.

## Introduction

The first suggestive evidence of benefit to patients with cancer from aspirin was reported over 50 years ago. Studies of animals with cancer showed that aspirin is associated with a reduction in the development of metastases [1, 2]. Since then, despite the reporting of much further evidence on biological effects of aspirin, and the reporting of many studies on aspirin and survival, there is still uncertainty about the role of aspirin as a possible adjuvant treatment of patients with cancer.

A number of small and inadequate randomised trials have been reported [3–6] and the pooling of results from these gives a suggestive reduction of 9% in cancer deaths in the 722 patients with cancer who had been randomised to aspirin (hazard ratio (HR): 0.91; 95% confidence interval (CI): 0.79, 1.04) [7]. While this result is only suggestive, a trial which developed within the cohort of the US Physicians Health Study of cancer prevention by aspirin is more strongly supportive. Just over 500 subjects in the cohort developed cancer, and those who had been randomised to aspirin showed a reduction in cancer deaths (HR: 0.68, 95% CI: 0.52, 0.90) [8].

Another source of evidence on the range of cancers to which aspirin may be relevant comes from opportunistic long-term follow-up studies of patients who had been involved in early randomised trials of aspirin and vascular disease. In addition to reporting a subsequent reduction in cancer incidence, Rothwell *et al* [9] and Mills and Wu [10] showed that deaths from a wide range of cancers were reduced in subjects who had been randomised to aspirin, and furthermore, the occurrence of metastatic spread was reduced in a range of cancers, including colon, brain, liver, lung and ‘other or multiple sites’ [11].

A number of new *ad hoc* randomised trial have been set up to test aspirin treatment in a few cancers and results from these are awaited [12–15]. These, however, are testing aspirin in only a very few cancers – principally colon, but also breast and prostate – while the effects of aspirin on biological mechanisms relevant to cancer lead to the possibility of benefit in most, if not all cancers [16–18]. Indeed, because of its manifold effects on biological processes, Zhang *et al* [19] suggest that aspirin is ‘a master regulator of the hallmarks of cancer’.

The bulk of published evidence on aspirin and the treatment of cancer comes, however, from observational studies and in this report, we present the results of 118 published observational studies to test the hypothesis that aspirin is of benefit to a wide range of cancers and not just one or a few common cancers. We also present evidence that aspirin, relative to cancer and in comparisons with other cancer treatments, is a very safe drug.

## Methods

We conducted three consecutive systematic literature searches and meta-analyses of published observational studies of aspirin taken by patients with cancer. Detailed reports on the first two searches have been published [7, 20]. A description of the most recent search procedure is given in Supplementary File 1, and in Supplementary File 2 a brief description of each of the studies judged to be relevant in the most recent search is presented. Together the three searches covered up to March 2020.

Given that most of the available studies have been on the three common cancers: colon, breast and prostate, and in view of the fact that aspirin is being tested in randomised trials, we first present pooled evidence on aspirin and these three cancers. We then present evidence from 36 published reports of 15 other cancers, each of which has been examined in only one or a very few studies.

The procedures adopted followed the PRISMA guidelines throughout [21] and a full description of the search strategy is given in Supplementary File 1. In brief: each of the three systematic searches using keywords was conducted by AW and DM in MEDLINE and EMBASE. The searches were limited to human studies in peer-reviewed journals. Relevant studies were selected by two authors (PE and GM) if (a) the studied population comprised patients diagnosed with cancer; (b) aspirin appears to have been taken regularly after cancer diagnosis; (c) the studies were randomised trials, case–control studies or cohort studies. Reference lists of the relevant studies identified were searched for other relevant reports. At least one author on each selected paper in all three searches was written to and asked specifically about gastrointestinal (GI) bleeding in the patients included in their study, together with appropriate further questions.

Data on cancer deaths and deaths from all-causes in the most recent search to March 2020 are listed in Supplementary File 3, first for studies that had expressed association as HRs, followed by studies which had used odds ratios (ORs), risk ratios (RRs) or percent survival. The methodological quality of the studies was assessed and graded independently by two authors (AW and PE) using the Newcastle–Ottawa Scale [22]. We have also added to each paper listed in Supplementary File 3 a comment as to the level of certainty that aspirin had been taken – or had not been taken – throughout follow-up.

Most of the risk estimates reported by the authors were expressed as HRs, and these and their 95% CI were taken from the original articles and log-transformed to obtain the estimate of the treatment effect (TE). The standard errors (seTE) were determined by subtracting the lower log-transformed CI boundary from the upper log-transformed CI boundary and dividing this by 3.92 (2*1.96). Where HRs were not reported, ORs, RRs and their 95% CI, or number of events among patients taking aspirin and those not on aspirin, were taken from the original articles. ORs and exact 95% CIs were calculated where needed, and all were then log-transformed for meta-analysis.

Summary risk estimates of random effects models are shown as forest plots in Supplementary File 4. HR meta-analyses were conducted using the meta package, version 4.13.0 in R 4.0.2, open source. Analysis with the metagen function used sm = HR for the underlying summary method and the DerSimonian–Laird method [23] was used to estimate the between-study variance (τ and τ^2^). Meta-analyses of the reports as ORs were conducted using Stata/SE 16.1, and used the restricted maximum likelihood method to estimate the between-study variance and these are shown as forest plots in Supplementary File 5.

Finally, funnel plots were constructed and estimates of the probability of publication bias were derived. The forest plot added trim and fill which mirrored the studies followed by a cumulative forest plot based on decreasing standard error. This was only undertaken on a minimum of 10 papers hence there is only one examination for OR. These are all shown in Supplementary File 6.

## Results

Three systematic literature searches on the topic of this report were conducted by the authors: in 2016 [7], in 2018 [20] and in 2020 up to March 2020 (Supplementary File 1). In each report, there are two outcomes, death from cancer and death from any cause, almost all of which have been presented as HRs. The new studies are described in Supplementary File 3 and their results are listed and pooled in Supplementary File 4. Some of the deaths have however been reported as OR, relative risk, etc., and all these have been converted to ORs. These ORs are presented separately from the HRs in Supplementary File 3 and are listed and pooled in Supplementary File 5. Some results however have been presented as additional survival in months or years, or during defined periods of time, such as 5 years. These are mentioned in the text, but do not appear in any table or Supplementary file.

In addition, we were concerned about undesirable side effects of the aspirin and in addition to abstracting relevant data from the published reports, following each of the three searches we wrote to an author of every report, asking for details of any unwanted side effect and in particular bleeding attributable to aspirin. A few authors supplied evidence on bleeding further to that in their published report, and these details are quoted in the text.

**Figure 1.**
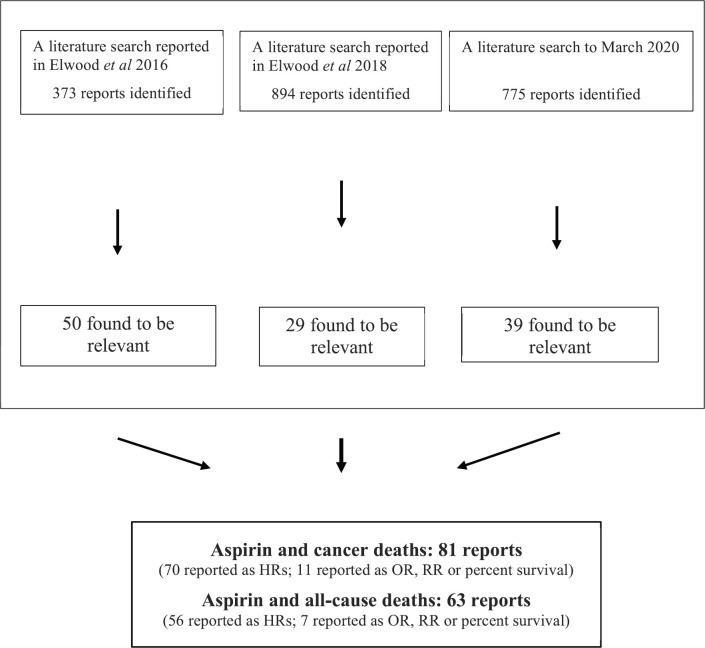
Flow diagram describing the findings of the three systematic literature searches.

Figure 1 describes the findings of the three searches.

### Mortality

For colon cancer mortality, our three literature searches identified a total of 24 studies in which the association with aspirin was reported as HRs. Together, these give a pooled HR of 0.72 (95% CI: 0.63, 0.82), and a single report showed an OR (OR of 0.78 (0.66, 0.93) (Table 1 and Supplementary File 4). For all-cause mortality, 20 studies of colon cancer reported HRs, giving a pooled association with aspirin of 0.83 (95% CI: 0.75, 0.92) and a single HR reported an OR of 0.78 (0.65, 0.92) (Table 1 and Supplementary File 4)

For breast cancer mortality, 13 studies reported as HRs and these give a pooled HR: 0.84 (0.72, 0.98). Four further studies give pooled OR: 0.75 (0.36, 1.57). For all-cause mortality in the breast cancer studies, nine reports give a pooled HR of 0.94 (0.70, 1.25).

For prostate cancer mortality, the pooling of 15 studies gives an HR of 0.89 (0.78, 1.02) and there was one study with an OR of 1.02 (0.78, 1.34). For all-cause mortality in prostate cancer reports, seven studies give an HR of 1.00 (0.78, 1.27) and in one the OR is 1.06 (0.94, 1.19).

For cancers other than colon, breast and prostate, the supplementary files list ‘other’ cancers: nasopharynx [96, 102], GI cancers, including oropharynx, stomach, oesophagus and rectum, [41, 88, 97, 106, 121, 124], liver [93, 103], gallbladder in four parts [101], pancreas [125], bladder [98, 112, 114], ovary [81, 83–86, 113], endometrium [87, 89], head & neck [88, 90–92, 104, 123], lung [82, 94, 108, 122], leukaemia [79], glioma [100], melanoma [99] and two [39, 80] present a mixture of cancers.

Not all the estimates of association in these reports of ‘other’ cancers are significant at *p* < 0.05. However, only three are consistent with an oropharynx possible harmful effect of aspirin, having a confidence limit which includes 1, but none of the three is significant at *p* < 0.05 with both confidence limits above 1.

Together, these reports of ‘other’ cancers give a pooled HR for cancer mortality of 0.79 (0.70, 0.88) in 18 studies and a pooled OR of 0.49 (0.26, 0.95) in five studies. All-cause mortality in 21 of these other cancers gives a pooled HR of 0.67 (0.60, 0.75) in 21 studies and the five studies that did not report HRs give a pooled OR of 0.47 (0.26, 0.83).

The forest plots of all these data are shown in Supplementary Files 4 and 5, and Table 2 brings together all the available data on cancer deaths and on all-cause mortality.

A number of authors give estimates of the association with aspirin in terms of the duration of the additional survival in patients taking the drug. Thus, Albandar [117] who followed 174 US veterans with colorectal cancer to death reported that the median survival of patients taking aspirin was 941 versus 384 days in those not taking aspirin. Several papers record an increased survival associated with aspirin taken by patients with liver cancer: in one 18 months additional survival [93]; in another 6% more patients survived 10 years with aspirin after diagnosis [103], and the median overall survival period after embolisation was longer for patients taking aspirin (57 versus 23 months) [119]. In a study of endometrial cancer, 91% of patients taking aspirin survived 10 years compared with 81% of patients not on aspirin [87]. In a study of patients with lung cancer, patients on aspirin survived 1.69 and only 1.02 years if not on aspirin [96]. In a study of pancreatic cancer, the 3-year survival was reported to be 61% in patients taking aspirin versus 26.3% in patients not taking aspirin [118], and finally, the 3-year survival of US veterans with head and neck cancer was 79% in those taking aspirin, compared with only 56% of those not taking aspirin [92].

Using a different approach, a group in Liverpool used data for over 44,000 patients with colon cancer to derive a predictive equation which relates a number of factors present at diagnosis to survival [45]. Entering the details for a non-diabetic man aged 70 with colon cancer into the predictive formula, the inclusion of aspirin taking increases the estimate of survival by about 5 years, and for a woman, about 4 years.

Finally, as a test of the hypothesis posed in this report, we compared the association of aspirin and cancer mortality in the 15 less common cancers with cancer mortality in colon cancer. In this comparison, we use colon cancer mortality as the ‘gold estimate’ of the effect of aspirin on the grounds that the effect of aspirin has been more thoroughly investigated in colon cancer, than in any other cancer; colon cancer is the only cancer for which the UK National Institute for Clinical Excellence has given a limited recommendation for aspirin, [120] and the U.

S. Preventive Services Task Force and other professional bodies give guidance for the use of aspirin in colon cancer [121].

This comparison shows:

**Colon Cancer mortality:**

24 studies give a pooled** HR: 0.72** (95% CI: 0.63, 0.82),

No significant publication bias z = −0.7276, *p* = 0.4668.

**Cancer mortality in less common cancers**

18 studies give a pooled** HR: 0.79** (95% CI: 0.70, 0.88),

Significant publication bias z = −2.8110, *p* = 0.0049.

### Bleeding

A search for evidence on bleeding, and fatal bleeding attributable to aspirin was made, and this included writing to the corresponding author on all the 118 papers included in the three searches. Many of the studies however had been based on recorded data, with no direct contact with the patients involved, and authors of such reports had little or no knowledge about bleeding in the patients they described.

Many of the authors reported the expected excess in GI bleeding in the patients on aspirin. However, only a very few reported fatal bleeds. In one study, 3% of the patients taking aspirin and 3.2% of those not taking aspirin had had a fatal bleed [40]. Tsoi *et al* [49], who studied a cohort of over 18,000 patients with colon cancer reported that deaths of aspirin users who developed GI bleeding were 0.40%, compared with 0.36% of the patients not taking aspirin. A study of patients with liver cancer treated by transarterial chemoembolisation reported that six patients in the aspirin group and seven patients in the non-aspirin group died because of upper GI bleeding [93]. One paper makes mention of the reduction in bleeding in patients who took a PPI along with the aspirin (OR: 0.85; 0.80, 0.91) [49]. All the references to bleeds relate to GI bleeds and no author made mention of cerebral bleeding.

## Discussion

This report provides both confirmatory and new evidence on the benefit of aspirin in reducing mortality in patients being treated for cancer. Replication is an important procedure in science and the present study confirms the findings of our first report with 50 studies [7], and our second report with 29 studies [20]. The present study is a further replicate with 39 new observational studies.

The meta-analyses we now present are all based on pooling of the data provided by 118 observational studies comprising about a quarter of a million patients with cancer who were recorded as taking aspirin. This reveals that aspirin taking is associated with a reduction of cancer deaths of about one fifth in a range of 18 cancers (HR: 0.79 (0.73, 0.84) in 70 observational studies and OR: 0.67 (0.45, 1.00) in 11 studies (Table 2 and Supplementary Files 4 and 5). The effect of aspirin on all-cause mortality is closely similar (HR: 0.80 (0.74, 0.86) in 56 observational studies and OR: 0.57 (0.36, 0.89) in 7 studies). A reasonable interpretation of these results is – that at any time after a diagnosis of a wide range of different cancers, about 20% more of the patients who take aspirin are likely to be alive, compared with patients not taking aspirin.

The evidence of publication bias throughout this work is a most important issue. Bias due to the selective publication of positive findings for aspirin was expected, and for some of the pooled results the magnitude of this bias is greater than could be reasonably expected in chance grounds alone (Supplementary File 6). While conclusions drawn from these 118 papers have, therefore, to be cautious, the evidence is strengthened by the absence of significant bias at *p* < 0.05 for the data for colon cancer. It is also encouraging that the trim and fill analysis on the less common cancers maintained the beneficial TE for both cancer specific mortality and all-cause mortality.

### Bleeding

A bleed, either GI or intra-cerebral, is a crisis for a patient, but the seriousness of a bleed attributable to aspirin should be evaluated against the likely benefits attributable to its use and furthermore the severity of the additional bleeds attributable to aspirin should be considered and not just their frequency [122, 123]. In relation to the treatment of cancer, our examination of the 118 reports gives a considerable degree of reassurance on aspirin, and particularly on the most serious bleeds. It is of relevance that most of the patients appear to have been taking low-dose aspirin primarily for cardiovascular protection.

Low-dose aspirin is however associated with additional GI bleeds in between 0.8 and 5.0 patients per 1,000 person years aged 50–84 years in the general population [124]. This represents an increase above spontaneous GI bleeding of between about 50% [125] and 90% [121]. It is important to note that these increases imply that only one in every two or every three bleeds that occur in patients taking low-dose aspirin is likely to be truly attributable to the aspirin, the other bleeds being spontaneous and nothing to do with aspirin.

The most serious bleeds are those that lead to death and our concern on this led us, early in our investigations of aspirin and cancer, to conduct a careful evaluation of fatal bleeding [126] A meta-analysis based on 11 randomised trials showed that the additional bleeds attributable to aspirin are less serious than spontaneous bleeds and are seldom, if ever fatal (relative risk of death: 0.45; (0.25, 0.80), and the risk of a fatal bleed in the totality of subjects randomised to aspirin, relative to subjects randomised to placebo was RR: 0.77 (0.41, 1.42). As we reported in our overview [126], others have reported similar findings of a reduction in the proportion of fatal bleeds in patients taking aspirin [9, 127–130].

Findings on bleeding in the recent ASPREE trial of prophylactic aspirin are of interest as more than 19,000 subjects with a median age of 74 years were followed for 5 years. Eighty-nine subjects randomised to aspirin, or 1.9 in every 1,000 experienced a bleed each year, compared with 48 bleeds, or just over 1.1 per thousand per year in those not taking aspirin. Granted this was not a trial of aspirin treatment, but it is of relevance to the safety of the drug that only two fatal bleeds occurred, and neither was in a subject taking aspirin [131].

The most serious side effect of aspirin, intra-cerebral bleeding, is fortunately rare [132], and no author in our literature review mentioned cerebral bleeding within the patients they followed. The risk associated with aspirin is estimated to be around 1.39 (95% CI: 1.08, 1.78) [125] equivalent to one or two additional haemorrhagic strokes per year in every 10,000 subjects [133].

Hypertension is the major factor in haemorrhagic stroke and in one major overview of randomised trials there was a doubling of cerebral haemorrhages for a rise of 20 mmHg in blood pressure (RR: 2.18; 95% CI: 1.65, 2.87) [127]. The relevance of hypertension was further highlighted in a trial of aspirin based on 20,000 patients with hypertensive disease, all of whom were adequately treated with anti-hypertensive drugs. There were no additional cerebral bleeds attributable to aspirin: the same number of patients on aspirin experienced cerebral bleeds (19 patients) as those on placebo (20 patients) [133].

## Strengths and limitations of this study

In addition to the risks of publication bias as detailed above, a most important limitation is that almost all the evidence we present are from observational studies. A number of randomised trials of therapeutic aspirin are in progress but these focus entirely on either one, or a few of the common cancers: colon [12–15], breast [12, 14] and prostate [12, 15]. Our concern, however, is for all cancers and not one or a few cancers, and as others have pointed out many of the actions of aspirin on cancer development, growth and metastatic spread, appear likely to be relevant to a wide range of cancers [6–19].

It is important to note that amongst the uncertainties in these observational studies, two uncertainties appear to stand out in their probable relevance to every observational study, and to the possible size of their effects. These are: first: uncertainties about the classification of patients with regard to continuous aspirin taking, and uncertainties about the non-taking of aspirin by the ‘controls’, and secondly, co-morbidity in the patients taking aspirin.

Few authors give reassurance about continued aspirin taking during follow-up, and no authors comment on the possibility of ‘contamination’ of control subjects starting to take ‘over the counter’ aspirin during the follow-up. An additional column in Supplementary File 3 lists quotations from the papers reviewed and these show that most authors assumed that if there is evidence of aspirin taking at the time of diagnosis, it can reasonably be assumed that aspirin taking was continuous during follow-up. Thus, ‘Low-dose aspirin use was defined as a minimum of one filled prescription after cancer diagnosis’ [89] and another: ‘the patients were receiving aspirin from diagnosis to at least 1 year after treatment initiation’ [90]. One author pointed out however that ‘the inverse association with aspirin appeared to be only among men who reported using aspirin regularly’, [76] and another noted that a reduction in mortality was ‘notably among patients filling prescriptions for a large quantity of low dose aspirin tablets during the (follow-up) period [77]. Another author found that prescribed aspirin alone was not associated with decreased mortality, but when OTC aspirin was added, a large reduction was detected [39].

A recent study by a group in Dublin examined the influence of approaching death on end-of-life aspirin use in patients with breast or colorectal cancer. They found that the use of aspirin declined ‘considerably’ during the 2 years before death, and at the time of death rates of aspirin use had dropped from around 60% to around 20% for colorectal cancer and from around 80% to around 45% for breast cancer [134].

The only comment about aspirin taking by control subjects comes from an overview of 12 studies in which the authors state that the pooled survival in patients on aspirin was only HR: 0.96 (0.88, 1.04) but if non-aspirin taking was more tightly defined as less than once per week, the HR was 0.89 (0.82, 0.98) [135].

The other important limitation is confounding by co-morbidity. Many authors mention that the aspirin takers in their study were older than the control patients not on aspirin. While this can be adjusted for statistically, the fact that a number of studies state that most of the patients who were taking aspirin were doing so because of a prior vascular event or prevalent vascular disease. Clearly, the morbidity that had led some of the patients to take aspirin can have eroded any benefit achievable by aspirin and while many of the papers mention this, few give details.

Yet a further limitation arises from possible miscoding of the causes of death in these studies. In the SEER programme on mortality in patients with cancer in the USA, it was found that 11% of cancer deaths had been attributed to vascular disease [136]. Any such miscoding will lead to an underestimate of the reduction in cancer deaths associated with aspirin.

The very broad range in the estimates of effect of aspirin leading to high heterogeneity estimates in our meta-analyses is worrying, and some of the differences between studies seem to defy any reasonable explanation. And yet, this was predicted from the beginning of the work on aspirin treatment [**7**]. There are many biases and sources of possible differences between the series of patients in the various studies, including differences in age and social factors, differences in other treatments and in general clinical management [41, 48]. Then there are possible differences in consistency of aspirin taking and the differences in co-morbidity already mentioned. Both poor aspirin taking and co-morbidity in patients taking aspirin will increase heterogeneity, and are probably inevitable in a series of studies such as we present. On the other hand, it seems unlikely that such differences could account for the overall benefits we find to be associated with aspirin taking.

## Conclusions

We judge that the body of evidence now available on the efficacy and the safety of aspirin justifies its use as an adjunct treatment in a wide range of cancers. Clinical care includes an ethical imperative for shared decision making [137] and we, therefore, believe that doctors should present, and patients with cancer should be encouraged to raise the topic of aspirin taking with their doctors. At the same time, we stress that aspirin is not a possible alternative to any other treatment, although in poorer countries aspirin could be one of very few, or perhaps the only acceptable treatment on the grounds of cost and availability [138].

Further research into aspirin and cancer would clearly be of great value, and studies including observational and randomised trial should be encouraged, especially if focused upon one of the less common cancers.

## Conflicts of interest

The author(s) declare that they have no conflict of interest. All the authors have read the paper and agree with its content.

## Funding

No special funding was obtained for any of the work described in this paper. Julieta Galante was supported by the National Institute for Health Research (NIHR) Applied Research Collaboration East of England. The views expressed are those of the author(s) and not necessarily those of the NHS, the NIHR or the Department of Health and Social Care.

## Figures and Tables

**Table 1.** Summary of Eggers test for bias and of trim and fill analysis.

	Egger’s test	Effect before trim and fill	Results robust after trim and fill?	Confidence interval after trim and fill
Colon cancer mortality *n*= 24	No bias 0.654	There was an effect 0.72 (0.63, 0.82)	Yes (no cases trimmed)	same
Colon All Cause mortality *n*=20	Bias 0.007	There was an effect 0.83 (0.75, 0.92)	No	(0.87, 1.07)
Other Cancers cancer mortality *n*=18	Some Bias 0.010	There was an effect 0.79 (0.70, 0.88)	Yes	(0.77, 0.98)
Other cancers all cause mortality *n*=21	Bias 0.022	There was an effect 0.67 (0.60, 0.75)	Yes	(0.66, 0.83)
Breast cancer mortality *n*=13	Some Bias 0.089	There was an effect 0.84 (0.72, 0.98)	No	(0.85, 1.19)
Breast cancer all cause mortality *n*=9	Small numbers No Bias 0.977	There was no effect 0.94 (0.70, 1.25)	N/A, no cases trimmed N/A no effect before trim and fill	same
Prostate cancer mortality *n*=15	No Bias 0.169	There was no effect 0.89 (0.78, 1.02)	N/A no effect before trim and fill	(0.87, 1.14)
Prostate cancer all cause mortality *n*=6	N/A	There was no effect 1.00 (0.78, 1.27)	N/A no effect before trim and fill	(0.88, 1.43)
All cancers combined cancer mortality *n*=92	No Bias 0.428	There was an effect 0.79 (0.73, 0.84)	Yes	(0.79, 0.91)
All cancers combined all cause mortality *n*=56	Bias <0.001	There was an effect 0.80 (0.74, 0.86)	No	(0.87, 1.02)

**Table 2.** A summary of the overall findings of the association between aspirin taking and mortality in 106 reports.

Group	Pooled estimates (Random effects model)			
	Cancer mortality		All-cause mortality	
	Numbers of studies	HRs (95% CIs) ORs (95% CIs)	Numbers of studies	HRs (95% CIs) ORs (95% CIs)
Colon Cancer	24 HRs One OR	0.72 (0.63, 0.82) 0.78 (0.66, 0.93)	20 HRs One OR	0.83 (0.75, 0.92) 0.78 (0.65, 0.92)
Breast cancer	13 HRs 4 ORs	0.84 (0.72, 0.98) 0.75 (0.36, 1.57)	9 HRs No OR	0.94 (0.70, 1.25) -
Prostate cancer	15 HRs one ORs	0.89 (0.78, 1.02) 1.02 (0.78, 1.34)	7 HRs One OR	1.00 (0.78, 1.27) 1.06 (0.94, 1.19)
15 other cancers^a^	18 HRs 5 ORs	0.79 (0.70, 0.88) 0.49 (0.26, 0.95)	21 HRs 5 ORs	0.67 (0.60, 0.75) 0.47 (0.26, 0.83)
Total 18 cancers	70 HRs 11 ORs	0.79 (0.73, 0.84) 0.67 (0.45, 1.00)	56 HRs 7 ORs	0.80 (0.74, 0.86) 0.57 (0.36, 0.89)

a Other cancers: Nasopharyngeal, Oropharyngeal, Oesophagus, Gastric, Rectal, Liver, Gallbladder, Pancreas, Bladder, Endometrium, Ovary, Glioma, Head & Neck, Lung, Melanoma

## Supplementary File 1. Search strategy.

**SUPPLEMENTARY FILE 1**

**ASPIRIN AND CANCER SURVIVAL IA systematic review and meta-analyses of 118 observational studies of aspirin and 18 cancers**

Peter C Elwood, Gareth Morgan, Christine Delon, Majd Protty, Julieta Galante, Janet Pickering, John Watkins, Alison Weightman, Delyth Morris **Search strategy**

**Search strategy developed using the following search filters for study design.**

- Observational studies: SIGN filter (http://www.sign.ac.uk/methodology/filters.html#obs)
- Randomised controlled trials: Cochrane highly sensitive search filter (http://handbook.cochrane.org/chapter_6/box_6_4_c_cochrane_hsss_2008_sensmax_ovid.htm)

**MEDLINE and Medline in Process (searched 11 March 2020)**

1. randomized controlled trial.pt.
2. controlled clinical trial.pt.
3. randomized.ab.
4. placebo.ab.
5. drug therapy.fs.
6. randomly.ab.
7. trial.ab.
8. groups.ab.
9. or/1-8
10. Epidemiologic studies/
11. exp case control studies/
12. exp cohort studies/
13. Case control.tw.
14. (cohort adj (study or studies)).tw.
15. Cohort analy*.tw.
16. (Follow up adj (study or studies)).tw.
17. (observational adj (study or studies)).tw.
18. Longitudinal.tw.
19. Retrospective.tw.
20. Cross sectional.tw.
21. Cross-sectional studies/
22. or/10-21
23. 9 or 22
24. exp animals/ not humans.sh.
25. 23 not 24
26. Exp neoplasms/
27. (cancer* or malign* or tumour* or tumor*).tw
28. 26 or 27
29. Aspirin/
30. (aspirin* or “acetylsalicylic acid”).tw
31. 29 or 30
32. 25 and 28 and 31
33. Limit 32 to ed”20180529-20200311”

**EMBASE (searched 11 March 2020)**

1. Random*.tw
2. Clinical trial*.mp
3. Exp health care quality/
4. placebo.ab.
5. or/1-4
6. clinical study/
7. case control study/
8. family study/
9. longitudinal study/
10. retrospective study/
11. prospective study/
12. Cohort analysis/
13. (Cohort adj (study or studies)).mp.
14. (Case control adj (study or studies)).tw.
15. (follow up adj (study or studies)).tw.
16. (observational adj (study or studies)).tw.
17. (epidemiologic* adj (study or studies)).tw.
18. (cross sectional adj (study or studies)).tw.
19. or/6-18
20. 5 or 19
21. exp animal/ not human.sh.
22. 20 not 21
23. Exp neoplasms/
24. (cancer* or malign* or tumour* or tumor*).tw
25. 23 or 24
26. Aspirin/
27. (aspirin* or “acetylsalicylic acid”).tw
28. 26 or 27
29. 22 and 25 and 28
30. Limit 29 to “year = Aug 2017 to March 2020”

## Supplementary File 2. Details of the new studies included in the review.

**A systematic review and meta-analyses of 118 observational studies of aspirin and 18 cancers**

Peter C Elwood, Gareth Morgan, Christine Delon, Majd Protty, Julieta Galante, Janet Pickering, John Watkins, Alison Weightman, Delyth Morris

**SUPPLEMENTARY FILE 2**

**Description of papers identified in the 2021 literature search.**

**Those which reported Hazard Ratios (HRs) are shown first, followed by those which reported RRs, OR, etc.**

**Details of papers identified in the literature searches in 2016 are given in Elwood *et al* [7] and those identified in the 2018 search are given in Elwood *et al* [20].**

**1. Studies in which results are reported as hazard ratios**

**Table:**

Source	Organ	Type of study and No.	Number in the cohort Length of follow-up	No. of cancer deaths	Comment	N-O score
Gray *et al* [43]	Colon	Retrospective cohort	1,539 patients FU 5–9 years	212 deaths	Data on aspirin and PIK3CA status concludes that restriction of aspirin to patients with the mutation should be unreasonable	**8**
Gray *et al* [48]	Colorectal	Retrospective cohort	8,391 patients FU 3.6 years	1,064 cancer deaths	Above paper on patients in N.I., this one closely similar with a cohort of patients in Scotland	**8**
Murphy *et al* [47]	Colon	Prospective Cohort study	95/296 FU 110 months	74/117 events	Data given on PIK3CA	**4**
Tsoi *et al* [49]	Colorectal	Retrospective cohort	5118/13,336 FU 14 years	9,026 deaths	Marginal increase in fatal bleeding in aspirin users	**8**
Frisk *et al* [60]	Breast	Swedish Population cohort	4,091/21,531 FU 3.8 years	*241/834*	Aspirin associated with a reduction in deaths in patients with stage I cancer	**8**
Bens *et al* [59]	Breast contralateral	Danish Population cohort	1,444 4.8 years	*n.a.*	Contralateral breast cancer in survivors of breast cancer	**8**
Strasser-Wippl *et al* [61]	Breast	Prospective cohort on aspirin. RCT of two other drugs	476/1,733 4.1 years	125 deaths from any cause	Complex design of study. Random allocation of two drugs and after 4 years one of these stopped	**4**
Wang *et al* [62]	Breast	Population based cohort	1,442 women FU 18 years	*n.a.*	Effect of aspirin greater when pattern of use taking into account	**8**
McCarthy *et al* [63]	Breast	Retrospective cohort	267 FU 7 years	*n.a.*	Exclusion of women with negative hormone receptor Relates tumour PIK3CA interaction to clinical outcomes only 70% on aspirin	**6**
Skriver *et al* [77]	Prostate	Nationwide cohort study	29,136 patients FU 7.5 years	7,633 deaths	Reduction associated with aspirin was notably among patients filling scripts for a large quantity of aspirin tablets	**8**
Hurwitz *et al* [76]	Prostate	Prospective cohort	97 men FU up to 25 years	97 cancer deaths	Association of deaths with aspirin…’ appeared only to be among men who reported using aspirin regularly	**8**
Prause *et al* [78]	Prostate	Prospective cohort	789/**3,525** FU 9.6 years	Only 3 deaths from prostate cancer	PSA levels lower in ASA users	**5**
Frouws *et al* [41]	Gastro-intestinal	Retrospective cohort	13,715	5,138	Data given for oesophagus, stomach, pancreas, liver, colon and rectum deaths	**8**
Spence *et al* [97]	Oesophagus Stomach	Retrospective Cohort study Retrospective Cohort study	4,654 FU 0.5–17.2 years 3833	3240 cancer deaths 2390 cancer deaths	Separate patients with oesophageal cancer and other with gastric cancer, within the same cohort Some uncertainty about aspirin taking long term	**7**
Chuang *et al* [96]	Small cell lung cancer	Retrospective cohort study	53,344 and 6,986 on aspirin	n.a.	**Reported as median survivals. In response to an email a hazard ratio was supplied by the author**	**5**
Erickson *et al* [95]	Lung	? Prospective cohort	1220/1,634 FU 6 years	n.a.	1,408 Afro-Americans and 1,446 Euro-Americans	**8**
McMenamin *et al* [82]	Lung	Retrospective cohort	3,635 patients	n.a.	Associations were comparable by duration of use of aspirin	**8**
Beeghly-fadiel et.al. [81]	Ovary	Retrospective cohort	207/940 n.a.		Non-aspirin NSAIDs had a similar reduction to that of aspirin	**7**
Merritt *et al* [85]	ovary	Prospective US Nurses Hlth 1 and 2	964	512 cancer deaths	Pts who became recent users of ASA (HR 0.44 (0.26, 0.74))	**8**
Verdoot *et al* [86]	ovary	Nationwide cohort	4,117 FU 3.6 years	242/1,661	Danish population wide study	**8**
Lumley *et al* [92]	Head And neck	Retrospective cohort	84/245 FU 31 months		Aspirin users more likely to have early stage disease Aspirin takers followed up for one year longer than non aspirin	**4**
Hedberg *et al* [91]	Head and Neck	Prospective cohort	357 PIK3CA positive patients		Study limited to PIK3CA positive patients 93% of NSAIDs was aspirin and 73% took aspirin exclusively	**8**
Li *et al* [93]	liver	Case–control retrospective	46/60 FU 6 years+	29 on ASA 34 no ASA	18 months extra survival on ASA Six fatal bleeds on aspirin, 7 in non aspirin	**8**
Simon *et al* [103]	Liver	Retrospective Cohort	14,205/36,070 FU 8 years	10% in ASA 18% non ASA	All pts had had Hepatitis 5-15 years previously No difference in bleeding on ASA and no ASA	**8**
Pretzch *et al* [118]	pancreas	Retrospective study	18/64 FU 20 months	18 patients on ASA 64 not	Additional survival judged to be due to prolonged metastasis-free interval associated with aspirin taking	**8**
Lyon *et al* [98]	Bladder	Prospective cohort	461/600 FU 4.2 years	331 cancer deaths 111 other causes	No evidence of effect on distant metastases	**8**
Sperling *et al* [89]	Endometr.	Prospective cohort	6,694 FU 4.5 years	n.a.	A nationwide study	**7**
Jackson *et al* [101]	Gall bladder	Retrospective cohort	2,934 ?5 years	2,415 deaths	Higher comorbidity in aspirin users	**7**
Luo *et al* [102]	Nasopharanyx	Matched Case–control	113/448 patients 10 years	17/184 deaths	Propensity score matched control patients	**8**
Seliger *et al* [100]	Glioma	Retrospective cohort	45/547 FU 7.3 years	n.a.	Data on aspirin dose and duration mostly lacking	**7**
Rachidi *et al* [99]	Melanoma	Retrospective cohort	395/1127 2–16 years	n.a.	Inverse association between aspirin use and mortality in stage II and III, but not in stage I	**8**
Chae YK *et al* [79]	Chronic lymph.leuk.	Retrospective cohort	79/201		ASA plus statin – implies high co-morbidity? Compliance with ASA taking 81% ASA’	**6**

**Reports on ‘other’ cancers, described in our report published in 2016 (Elwood *et al*** [7]**):**

**Table:**

[83]	Nagle *et al* (2015)	Ovarian cancer
[108]	Fontaine *et al* (2010)	Lung cancer
[112]	Pastore *et al* (2015)	Bladder cancer
[80]	Chae *et al* (2013)	Mix of female cancers
[79]	Chae *et al* (2014)	Lymphocytic cancer
[88]	McFarlaine *et al* (2015)	Head and neck

**Reports on ‘other’ cancers, described in our report published in 2018 (Elwood *et al*** [20]**):**

**Table:**

[84]	Bar *et al* (2016)	Ovarian cancer
[87]	Matuso *et al* (2016)	Endometrium
[93]	Li *et al* (2016)	Liver cancer
[39]	Veitonmaki *et al* (2016)	Lung
[94]	Maddison *et al* (2017	Lung
[90]	Kim *et al* (2018)	Head and neck

**Table:**

Source	Organ	Type of study and No.	Number in the cohort Length of follow-up	No. of cancer deaths	Comment	N-O score
Din *et al* [109]	Colon	Case/control drawn from a trial cohort	234/526 FU n.a.	125/761	NSAIDs but data for aspirin given	
Reimers *et al* [105]	colorectal	Cohort study	178/784	69 deaths in users 380 in non-users	HLA Class 1 antigen amalgamated	**8**
Holmes *et al* [115]	Breast	Prospective study	27,426 FU 2.5 years	565/173	Daily aspirin associated with a reduction in deaths (HR 0.69) Less than daily associated with excess deaths (HR 1.43)	**8**
Bowers *et al* [110]	Breast	Prospective study of 440 women	159 users/281 not FU not available	Numbers no available	NSAIDS. 81% of patients took aspirin	**7**
Kwan *et al* [116]	Breast	Cohort of 2,292 women	FU 2.5 years	41/209 recurrent cancers	NSAIDs study. only 18 patients(7%) took aspirin post diagnosis	**8**
Murray *et al* [111]	Breast	Nested case–control study	1173/1173 FU 6.9 years	262/1435 cancer 1056/5697 deaths	Very imprecise definition of aspirin use by cases and use by controls	**4**
Cardwell *et al* [107]	Prostate	Case–control study	1,184/3,531 FU 4–12 years	616/568	Aspirin use obtained from GP records	**8**
van Staalduinen *et al* [106]	Oesophagus	Retrospective Cohort study	157/293 FU 0.83 years	*n.a.*		**6**
Baandrup [113]	Ovary	Case–control	3,741/50,576 FU 10 years	n.a.	A PhD thesis based on nationwide data	**6**
Rafei *et al* [104]	Head and neck	Retrospective cohort	86/246 FU 5 years	n.a.	Pts who filled more than one prescription, excluding refills, after diagnosis of HNC were considered ASA users	**7**
Gupta *et al* [114]	bladder	Prospective study	15/88 FU 18 months	recurrence	Very small numbers. High incidence (75%) of vascular disease. Also treated with BCG therapy	**4**
Chuang *et al* [96]	Naso pharynx	Matched case–control	1:3 matched 116/348	n.a.	Metastases free in 88% of ASA patients; 77% not on ASA	**3**
Luo *et al* [102]	Naso pharynx		113/452 FU 10 years	17/184	Data on cancer mortality stated as an HRData on all-cause death used for an OR	**8**

N-O score, Newcastle-Ottawa score based on eight points

## Supplementary File 3. Results: estimates of association: aspirin and mortality.

**A systematic review and meta-analyses of 118 observational studies of aspirin and 18 cancers**

Peter C Elwood, Gareth Morgan, Christine Delon, Majd Protty, Julieta Galante, Janet Pickering, John Watkins, Alison Weightman, Delyth Morris

**SUPPLEMENTARY FILE 3**

**Results of studies identified in the 2021 literature search.**

**Those which reported Hazard Ratios (HRs) are shown first, followed by those which reported RRs , OR, etc.**

**Results of studies identified in the literature searches in 2016 are given in Elwood *et al* [7] and those identified in the 2018 search are given in Elwood *et al* [20]**

**1. Studies in which results are reported as hazard ratios**

**Table:**

Source	Organ	ASA/none F-U	Evidence for continued aspirin taking	No of cancer deaths	HR 95% CI	No. of all-causes deaths	HR 95% CI	Comment	N-O scale
Gray *et al* [43]	Colon	146/534 5–9 years	Patients records, but not consistently recorded	40/172	**HR 0.69** 0.47, 0.98	64/534	**HR 0.76** 0.57, 1.03		**8**
Gray *et al* [48]	Colorectal	2,510/5,881 3.6 years	National prescribing records	335/729	**HR 1.17** 1.00, 1.36	600/1035	**HR 1.21** 1.07, 1.37	Cardiovasc deaths in pts on ASA **HR 1.63**	**8**
Murphy *et al* [47]	Colon	95/296 FU 110 months	‘75mg aspirin at diagnosis No check during follow-up	n.a.	**HR 0.63** 0.30,1.32	n.a.	**HR 1.26** 0.72, 2.21	Data given on PIK3CA	4
Tsoi *et al* [49]	Colorectal	5,118/13,336 FU 14 years	‘have been prescribed aspirin for at least 6 months.	2,073/13,336	**HR 0.59** 0.56, 0.62			Data on GI bleeding RR 1.09 on aspirin	**8**
Frisk *et al* [60]	Breast	4,091/21,418 FU 3.8 years	Evidence from National Prescribing register	241/834	**HR 0.99** 0.79, 1.23			**HR 0.53 ** (0.29, 0.96) In Stage 1	**8**
Bens *et al* [59]	Contralateral breast	52,723 FU 4.8 years	Two+ prescriptions in National Register	1,444	**HR 0.91** 0.75, 1.09				**8**
Strasser-W *et al* [61]	breast	476/1,733 FU 4.1 years	Patients taking more than 81 mg/day ineligible		**HR 1.48** 1.12, 1.96	56/110	**HR 1.68** 1.35, 2.61	Celecoxib and aspirin tested	**4**
Wang *et al* [62]	Breast	1,442 FU 18 months	Interview with pts 3 months > diagnosis	237 cancer deaths	**HR 0.87** 0.59, 1.29	597 all-cause deaths	**HR 1.21** 0.99, 1.48	Greater effect when pattern of aspirin taking allowed for	**8**
McCarthy *et al* [63]	Breast	54/213	inpatient and outpatient prescription records				**HR 1.04** (0.68, 1.54)		**6**
Skriver *et al* [77]	Prostate	7,163/21,973 5–7.5 years	2 or more scripts within one year	7,633 prostate deaths	**HR 0.95** 0.89, 1.01	13,208	**HR 0.95** 0.89,1.01 **+ HR 1.12** 1.05, 1.20	ASA dose related reductions	**8**
Hurwitz *et al* [76]	Prostate	6,594	4 interviews during follow-up	97 cancer deaths	**HR 0.58 ** 0.35. 0.95			Advanced disease at diagnosis selected	**5**
Prause *et al* [78]	Prostate	789/3,525 FU 9.6 years	Aspirin intake confirmed by two methods	3 cancer deaths		n.a.	**HR 1.46** 1.10, 1.94		**5**
Frouws *et al* [41]	Gastro intestinal	1008/13,715	Prescription records	1008/8278		362/4776	**HR 0.52** 0.44, 0.63		**8**
Spence *et al* [97]	oesophagus	4,654 FU 0.5–17.2 years	?one year Prescription records	3,240	**HR 0.98** 0.89, 1.09				**7**
	Gastric	3,833 FU 0.5–17.2 years	One year Prescription records	2,390	**HR 0.96** 0.85, 1.08				**7**
Chuang *et al* [96]	Non-small cell lung	3,487 Matched pairs	Prescription records	n.a.		5,918/5,149	**HR 0.79** 0.75, 0.83	Data obtained from the author	**6**
Erickson *et al* [95]	Non-small cell lung	1220/1,634 FU ?1–0 years	n.a.			150/209	**HR: 0.89** 0.74, 1.07	Combined EA/ AA data obtained from author	**5**
McMenamin *et al* [82]	Lung cancer	3,635	Prescription records		**HR 0.96** 0.85, 1.09				**6**
Beeghly-Fadiel *et al* [81]	ovary	207/940 ?	‘medication use determined from EMR’			Number of deaths n.a.	**HR 0.59** (0.46, 0.74)		**7**
Merritt *et al* [85]	ovary	/1,031	??? 3.8 years	458 deaths	**HR 0.68** (0.52, 0.89)				**8**
Verdoot *et al* [86]	ovary	4,117	‘filled prescriptions From Danish Registries’	242 cancer deaths	**HR 1.02** (0.87, 1.20)	70 /272	**HR 1.06** (0.77, 1.47)		**8**
Lumley *et al* [92]	Head and neck	84/329 FU 3 years	‘first script < a year of diagnosis and continued for at least a year’	Disease free	**HR 0.40** (0.21, 0.79)	3 year all-cause survival	**HR 0.51** (0.35, 0.76)		**4**
Hedberg *et al* [91]	Head and neck	358 patients with PIK3CA	script refill records and pt. self-reports	Patients with PIK3CA	**HR 0,23** (0.09, 0.62)	Patient with PIK3CA	**HR 0.31** (0.14, 0.69)	Only patients with PIK3CA mutation	**8**
Li *et al* [93]	liver	46/60 5 yeas F-U	100 mg administered continuously for 3m+’			46/46	**HR 0.50** (0.28, 0.89)		8
Simon *et al* [103]	liver	14,205/36,070 FU 8 years	‘first filled script For 90+ doses of ASA’	5,917/15,160	**HR 0.73** (0.67, 0.81)				**8**
Lyon *et al* [98]	bladder	461/600 4.2 years	‘aspirin users at the time of surgery’	331 cancer deaths	**HR 0,64** (0.45, 0.89)	442 deaths	**HR 0.70** (0.53, 0.93)		**8**
Sperling *et al* [89]	endometrium	6,694 4.5 years	‘a minimum of one filled prescription after diagnosis’	n.a.	**HR 1.10** (0.90, 1.33)			Survival with aspirin was age related	**7**
Jackson *et al* [101]	Gallbladder	605/2,934	‘aspirin was defined as one prescription or more’			GB 54/67 Chol. 123/140 Ampu. 26/38 Overlap 39/47	**HR 0.63** (0.48, 0.83) **HR 0.71** (0.60, 0.85 **HR 0.44** (0.26, 0.76) **HR 0.68** (0.50, 0.92)		**7**
Luo *et al* [102]	Nasopharynx	113/452 FU 10 years	Defined as ‘at least 180 days’	9/116	**HR 0.23** (0.12, 0.46)	17/184			**8**
Seliger *et al* [100]	Glioma	45/547 FU 7.3 years	‘dose and duration was mostly lacking’			Overall survival	**HR 0.71** (0.54, 0.93)		**7**
Rachidi *et al* [99]	Melanoma	395/1127 14-16 years	ASA use ‘based on scripts And medical records’			Overall survival	**HR 0.58** (0.45, 0.75)		**8**
ChaeYK *et al* [79]	Chronic lymph.leuk	71/242 9,8 months	‘concomitant aspirin’	Progression free survival	**HR 0.34** (0.18, 0.65)	Overall survival	**HR 0.40** (0.21, 0.79)	Results are for aspirin plus statins	**6**

**Papers on ‘other’ cancers, described in our report published in 2016 (Elwood *et al*** [7]**):**

Nagel *et al* [83] (2015)

Fontaine *et al* [108] (2010)

Pastore *et al* [112] (2015)

Chae *et al* [80] (2013)

Chae *et al* [79] (2014)MacFarlane *et al* [88] (2015)

**Papers on ‘other’ cancers, described in our report published in 2018 (Elwood *et al*** [20]**):**

Bar *et al* [84] (2016)

Matuso *et al* [87] (2016)

Li *et al* [93] (2016)

Veitonmaki *et al* [39] (2015)

Maddison *et al* [94] (2017)

Kim *et al* [90] (2017)

**2. Studies in which results are reported as RRs, ORs, etc.**

**Table:**

Source	Organ	ASA/none F-U	Evidence for continued aspirin taking	No of cancerdeaths	OR/RR 95% CI	No. of all-causes deaths	OR/RR 95% CI	Comment	N-O scale
Din *et al* [109]	Colorectal	354/526	‘We did not have info on aspirin after cases were diagnosed’	125, 761	OR 0.78 (0.65, 0.92)			Data also on NSAIDs	**6**
Reimers *et al* [105]	Colon	107,429	Users had at least on script For aspirin for 14 days				OR 0.78 0.65, 0.92		**8**
Holmes *et al* [115]	Breast	?/5,521 FU 48 months	No assessment of aspirin taking in 33%t	56/173	RR 0.36 (0.24–0.54)				**8**
Bowers *et al* [110]	Breast	159 /281	Only 81% of NSAIDs Was aspirin		OR 0.48 (0.22, 0.98)			NSAIDs, 81% was aspirin	**7**
Kwan *et al* [116]	Breast	270/2,292 FU 2.5 years	Use of aspirin or NSAID ‘at least 3 days/week’	41, 209	RR 1.09 (0.74, 1.61)			Other NSAIDs RR 0.56.	**8**
Murray *et al* [111]	Breast	262/1435 FU 5 years	‘at least one script for aspirin’ s	1,435 cancer deaths	OR 1.00 (0.71, 1.41)				**4**
Cardwell *et al* [107]	Prostate	1,184/3,531 FU 4-12 years	Aspirin taking was based on prescriptions in primary care	1559cancer deaths	OR 1.06 (0.92, 1.24)		OR 1.06 0.94,. 1.19		**8**
Fontaine *et al* [108]	Lung Cancer	412/1353 FU 7.5 years	Aspirin taking pre-op . No info post op.			180/564	HR 0.84 But no CIs		**6**
van Staald-uinen *et al* [106]	Oesophag.	105/157 FU 0.14 tears	‘at least one script for at least 14 days			74/129	RR 0.42 0.30, 0.57		**6**
Bandrup [113]	Ovary	3,741/50,576 FU 10 years	Overlapping Continuous scripts	n.a.	OR 0.56 (0.32, 0.96)				**6**
Rafei *et al* [104]	Head and neck	86/246 FU 5 years	‘number, date and dose of ASA scripts reviewed’	Number of deaths n.a	82% versus 43%;	Number of deaths n.a	72% versus 39%;		**7**
Pastore *et al* [112]	Bladder	98/287 1.5 to 6 years	‘particular attention to intake of aspirin’	42,98	OR 0.75 (0.45, 1.24)				**8**
Gupta *et al* [114]	Bladder	15/88FU 11 months	ASA taken for at least 3 months	recurrence	OR 1.00 (0.24, 4.16)				**4**
Chuang *et al* [96]	Naso Pharynx	116/348 FU 1–11 years	‘regular aspirin intake is not defined	4/19	85.9% versus 75.5% P = 0.30	24/43	87.7% versus 79.6%		**5**
Luo *et al* [102]	Naso Pharynx	113/452 FU 10 years	Defined as ‘at least 180 days’			17/184	62% versus 42.4%		**8**

Note: the final paper reported cancer mortality as an HR and all-cause as proportionate survival

## Supplementary File 4. Forest plots for estimates of aspirin and mortality as HRs.

**Supplementary file 4**

**ASPIRIN AND CANCER SURVIVAL I.**

**A systematic review and meta-analyses of 117 observational studies of aspirin and 18 cancers**

Peter C Elwood, Gareth Morgan, Christine Delon, Majd Protty, Julieta Galante, Janet Pickering, John Watkins, Alison Weightman, Delyth Morris

**Five forest plots of aspirin and deaths from cancer:** Colon, breast, prostate, other cancers, all cancers

**Five forest plots of aspirin and deaths from all-causes:** Colon, breast, prostate, other cancers, all cancers

## Supplementary File 5. Forest plots for aspirin and mortality as ratios other than HR.

**ASPIRIN AND CANCER SURVIVAL I.**

**A systematic review and meta-analyses of 114 observational studies of aspirin and 18 cancers**

Peter C Elwood, Gareth Morgan, Christine Delon, Majd Protty, Julieta Galante, Janet Pickering, John Watkins, Alison Weightman, Delyth Morris

**Supplementary file 5**

**Associations reported a ORs RRs and percentage survival all converted to ORs**

### Cancer-specific mortality

**All cancers**

**Other cancers**

**Breast cancer**

### All-cause mortality

**All cancers**

**Other cancers**

## Supplementary File 6. Estimates of publication bias.

### Publication Bias, Funnel plots

#### All cancers combined

**All cancers combined mortality**

13 cases added with Trim and fill

**Table:**

Regression Test for Funnel Plot Asymmetry model: mixed-effects meta-regression model predictor: standard error test for funnel plot asymmetry: *z* = −3.3121, *p* = 0.0009	Bias
Regression Test for Funnel Plot Asymmetry model: weighted regression with multiplicative dispersion predictor: standard error test for funnel plot asymmetry: *t* = −3.8370, df = 68, *p* = 0.0003	Bias
Intercept ConfidenceInterval *t p* Egger’s test -0.413 -1.393-0.567 -0.797 0.42811	No bias seen

**All Cancers Combined Cancer mortality:**

Trim and Fill and Cumulative forest plot ranked by SE with trim and fill.

Forest plot in order of SE with trim and fill mirrored studies added below.

Cumulative forest plot ranked by SE with Trim and fill at end.

Published value 0.79 (0.73, 0.84)

Results with trim and fill 0.85 (0.79, 0.91)

Results are robust with trim and fill.

**All Cancers Combined All-Cause Mortality**

19 cases added with Trim and fill

**Table:**

Regression Test for Funnel Plot Asymmetry model: mixed-effects meta-regression model predictor: standard error test for funnel plot asymmetry: *z* = −4.0797, *p* < 0.0001	Bias
Regression Test for Funnel Plot Asymmetry model: weighted regression with multiplicative dispersion predictor: standard error test for funnel plot asymmetry: *t* = −4.5330, df = 54, *p* < 0.0001	Bias
Intercept ConfidenceInterval *t p* Egger’s test -2.149 -3.325--0.973 -3.538 0.00084	Bias

**All Cancers Cancer All-Cause mortality:**

Trim and Fill and Cumulative forest plot ranked by SE with trim fill

Forest plot ranked by SE with trim and fill mirrored studies added below.

Cumulative forest plot ranked by SE with Trim and fill at end.

Published value 0.80 (0.74, 0.86)

Results with trim and fill 0.94 (0.87, 1.02)

Results are not robust with trim and fill.

#### Breast Cancer

**Breast Cancer Mortality**

Six cases added with Trim and fill

**Table:**

regtest(BreastResultREML, model = «rma», predictor = «sei») Regression Test for Funnel Plot Asymmetry model: mixed-effects meta-regression model predictor: standard error test for funnel plot asymmetry: *z* = -1.6897, *p* = 0.0911	Some Bias at *p*=0.1 level
Regression Test for Funnel Plot Asymmetry model: weighted regression with multiplicative dispersion predictor: standard error test for funnel plot asymmetry: *t* = -2.1738,* df* = 11, *p* = 0.0524	Some Bias at p=0.1 level
Intercept ConfidenceInterval t p Egger’s test -2.073 -4.229-0.0830000000000002 -1.865 0.08903	Some Bias at p=0.1 level

**Breast Cancer Mortality:**

**Trim and Fill and Cumulative forest plot ranked by SE with trim fill**

Forest plot ranked by SE with trim and fill mirrored studies added below.

Cumulative forest plot ranked by SE with Trim and fill at end.

Published value 0.84 (0.72, 0.98)

Results with trim and fill 1.00 (0.85, 1.19)

Results are not robust with trim and fill.

**Breast Cancer All-Cause Mortality**

Zero cases added with Trim and Fill

**Table:**

Regression Test for Funnel Plot Asymmetry model: mixed-effects meta-regression model predictor: standard error test for funnel plot asymmetry: *z* = −0.2868, *p* = 0.7743	No Bias seen But *n* too low
Regression Test for Funnel Plot Asymmetry model: weighted regression with multiplicative dispersion predictor: standard error test for funnel plot asymmetry: *t* = −0.2147, df = 7, *p* = 0.8361	No Bias seen But *n* too low
Intercept ConfidenceInterval t p Egger’s test -0.088 -5.772-5.596 -0.031 0.97639 Warning: The meta-analysis contains k = 9 studies. Egger’s test may lack the statistical power to detect bias when the number of studies is small (i.e., k < 10).	No Bias seen But *n* too low

**Breast Cancer All-Cause mortality:**

**Trim and Fill and Cumulative forest plot ranked by SE with trim fill**

Forest plot ranked by SE.

No Trim and fill as no cases added.

Cumulative forest plot ranked by SE. No Trim and fill.

Published value 0.94 (0.70, 1.25)

The same

Non-effect is robust with trim and fill.

#### Colon Cancer

**Colon Cancer Mortality,**

No cases added with Trim and fill

**Table:**

Regression Test for Funnel Plot Asymmetry model: mixed-effects meta-regression model predictor: standard error test for funnel plot asymmetry: *z* = −0.7276, *p* = 0.4668	No Bias
Regression Test for Funnel Plot Asymmetry model: weighted regression with multiplicative dispersion predictor: standard error test for funnel plot asymmetry: *t* = −0.7568, df = 22, *p* = 0.4572	No Bias
Intercept ConfidenceInterval *t p* Egger’s test 0.365 -1.203-1.933 0.454 0.65461	No Bias

**Colon Cancer mortality: Cumulative forest plot ranked by SE.**

Published value 0.72 (0.63, 0.82)

Colon results are robust.

**Colon Cancer All-Cause Mortality**

Eight cases added with Trim and fill

**Table:**

Regression Test for Funnel Plot Asymmetry model: mixed-effects meta-regression model predictor: standard error test for funnel plot asymmetry: *z* = −2.7423, *p* = 0.0061	Bias
Regression Test for Funnel Plot Asymmetry model: weighted regression with multiplicative dispersion predictor: standard error test for funnel plot asymmetry: *t* = −3.8054, df = 18, *p* = 0.0013	Bias
Intercept ConfidenceInterval *t p* Egger’s test -2.651 -4.415--0.887 -3.016 0.00742	Bias

**Colon Cancer All-Cause mortality:**

**Trim and Fill and Cumulative forest plot ranked by SE with trim fill**

Forest plot ranked by SE with trim and fill mirrored studies added below.

Cumulative forest plot ranked by SE with Trim and fill at end.

Published value 0.83 (0.75, 0.92)

Results with trim and fill 0.97 (0.87, 1.07)

Results are not robust with trim and fill.

#### Other Cancers

**Other Cancers Mortality**

Five cases added with Trim and fill

**Table:**

Regression Test for Funnel Plot Asymmetry model: mixed-effects meta-regression model predictor: standard error test for funnel plot asymmetry: *z* = −2.8110, *p* = 0.0049	Bias
Regression Test for Funnel Plot Asymmetry model: weighted regression with multiplicative dispersion predictor: standard error test for funnel plot asymmetry: *t* = −3.4563, df = 16, *p* = 0.0033	Bias
Intercept ConfidenceInterval *t p* Egger’s test -1.555 -3.319-0.209 -1.747 0.09976	Some Bias at *p*=0.1 level

**Other Cancers Cancer mortality:**

**Trim and Fill and Cumulative forest plot ranked by SE with trim fill**

Forest plot ranked by SE with trim and fill mirrored studies added below.

Cumulative forest plot ranked by SE with Trim and fill at end.

Published value 0.79 (0.70, 0.88)

Results with trim and fill 0.86 (0.77, 0.98)

Results are robust with trim and fill.

**Other Cancers All-Cause Mortality**

Seven cases added with Trim and fill.

**Table:**

Regression Test for Funnel Plot Asymmetry model: mixed-effects meta-regression model predictor: standard error test for funnel plot asymmetry: *z* = −1.9277, *p* = 0.0539	Bias at cutoff *p* < 0.1
Regression Test for Funnel Plot Asymmetry model: weighted regression with multiplicative dispersion predictor: standard error test for funnel plot asymmetry: *t* = −2.6072, df = 19, *p* = 0.0173	Bias
Intercept ConfidenceInterval *t p* Egger’s test -1.415 -2.591--0.239 -2.493 0.02209	Bias

**Other Cancers All-Cause Mortality**

**Trim and Fill and Cumulative forest plot ranked by SE with trim fill**

Forest plot ranked by SE with trim and fill mirrored studies added below.

Cumulative forest plot ranked by SE with Trim and fill at end.

Published value 0.67 (0.60, 0.75)

Results with trim and fill 0.74 (0.66, 0.83)

Results are robust with trim and fill.

#### Prostate Cancer

**Prostate Cancer Mortality**

Six cases added with Trim and fill.

**Table:**

Regression Test for Funnel Plot Asymmetry model: mixed-effects meta-regression model predictor: standard error test for funnel plot asymmetry: *z* = −2.0812, *p* = 0.0374	Bias
Regression Test for Funnel Plot Asymmetry model: weighted regression with multiplicative dispersion predictor: standard error test for funnel plot asymmetry: *t* = −3.1051, df = 13, *p* = 0.0084	Bias
Intercept ConfidenceInterval *t p* Egger’s test -1.244 -3.008-0.52 -1.456 0.16922	No Bias seen

**Prostate Cancer mortality**

**Trim and Fill and Cumulative forest plot ranked by SE with trim fill**

Forest plot ranked by SE with trim and fill mirrored studies added below.

Cumulative forest plot ranked by SE with Trim and fill at end.

Published value 0.89 (0.78, 1.02)

Results with trim and fill 1.00 (0.87, 1.14)

Non-effect is robust with trim and fill.

**Prostate Cancer All-Cause Mortality**

Two cases added with Trim and Fill

**Table:**

Regression Test for Funnel Plot Asymmetry model: mixed-effects meta-regression model predictor: standard error test for funnel plot asymmetry: *z* = −1.1081, *p* = 0.2678	No evidence of Bias but too few studies
Regression Test for Funnel Plot Asymmetry model: weighted regression with multiplicative dispersion predictor: standard error test for funnel plot asymmetry: *t* = −1.0350, df = 4, *p* = 0.3591	No evidence of Bias but too few studies
Intercept ConfidenceInterval *t p* Egger’s test -2.697 -9.361-3.967 -0.798 0.46976 Warning: The meta-analysis contains k = 9 studies. Egger’s test may lack the statistical power to detect bias when the number of studies is small (i.e., k < 10).	No evidence of bias

**Prostate Cancer All-Cause mortality:**

**Trim and Fill and Cumulative forest plot ranked by SE with trim fill**

Forest plot ranked by SE with trim and fill mirrored studies added below.

Cumulative forest plot ranked by SE with Trim and fill at end.

Published value 1.00 (0.78, 1.27)

Results with trim and fill 1.13 (0.88, 1.43)

Non-effect is robust with trim and fill.

Egger’s test

On ORs for 10+ studies of cancer-specific mortality meta-analysis for all cancers *Regression-based Egger test for small-study effects*

*Random-effects model*

*Method: REML*

* H0: beta1 = 0; no small-study effects*

*beta1 = -0.79*

*   SE of beta1 = 1.180*

*z = -0.67*

* Prob > |z| =* ***0.5011 (this is the p value)***

No evidence of publication bias.
